# 

**Published:** 1871-05

**Authors:** 


CONTENTS:

ORIGINAL CONTRIBUTIONS.

The Mystery of Life. An Address Delivered before the Alumni Association of the Chicago Medical College. By Jno. M. Woodworth, A.M., M.D., President, 257
Regulation of Prostitution—Results of the attempt in St. Louis, Missouri. By Edmund Andrews, M.D., ... 266
Antagonisms between Opium and Belladona. By Lyman Ware, M.D., Chicago, 267
A Brief Paper on the Effects of Alcohol and Tobacco, on the human System. By N. S. Davis, M.D., Chicago, Ill..... 274
Annual Report of the Necrologist of the Alumni Association of Chicago Medical College, ... 281

THE CLINIC.

Clinical Cases in Medical Wards of Mercy Hospital. By Prof. Davis. From Notes by S., ... 286

CORRESPONDENCE.

When Doctors Differ Where is Safety?... 294

OBITUARY.

Harrison Noble, M.D., Heyworth, Ill.,... 295
S. W. Noble, M.D., Bloomington, Ill.,... 296

SELECTIONS.

Scrofuloderma, ... 297
The Pathological Relations of the Gastric and Intestinal Tubules. By Austin Flint, M.D., ... 303
Chloral in Obstetrics, ... 307
Novel Mode of Controlling Hemorrhage, 308
A Case of Buccal Chancre, ... 309
Increasing Drunkeness in England, ..... 310
Cure of Old Ulcers by a Recently Devised Operation, ... 311
Investigations as to the Cause of the Great Gravity of Anthrax and Furuncles of the Face, ... 311
The Action of Urine on the Tissues, ..... 310
Chloral in Tetanus, ... 312
Treatment of Scarlet Fever and Scarlatinous Dropsy by Baths, ... 312
An Epidemic of Kine-Pox ... 312
Endemics and the Soil, ... 309
Subcutaneous Injections, ... 313
Ulcers a Cause of Bright's Disease, ..... 313
Bromide of Potassium in Uterine Fibroids, 313
Dermatological Conference, ... 314
Styptic Colloid, ... 314
The Internal Administration of Carbolic Acid in Pruritis and Prurigo, ... 314
Chloral for Sea-Sickness, ... 314
New Method of Reducing Dislocations of the Shoulder, ... 315
Chloral and Cerebral Disease, ... 315
Importance of Revaccination, ... 315
Incurability of Chronic Leucorrhea, ..... 315
Wrappers for Medical Journals, ... 315

BOOK NOTICES.

A Medico-Legal Treatise on Malpractice and Medical Evidence, ... 316
The Causation, Cure, and Treatment of Reflex Insanity in Women, ... 316
Modern Therapeutics: a Compendium of Recent Formulæ and Specific Therapeutical Directions, ... 317
Insanity and Its Treatment, ... 317
On the Wasting Diseases of Infants and Children, ... 317

EDITORIAL.

American Medical Editors and Medical Association Proceedings, ... 318
Wisconsin State Medical Society, ... 318
Poisoning from Belladonna.—Recovery,. 318
Illinois State Medical Society, ... 320
Treatment of Strychnia Poisoning by Bromide of Potassium, ... 321
Mortality for the Month of March, ...... 322
An Apothecary in Boston, ... 323

